# Consensus Report on *Shigella* Controlled Human Infection Model: Conduct of Studies

**DOI:** 10.1093/cid/ciz892

**Published:** 2019-12-09

**Authors:** Kawsar R Talaat, A Louis Bourgeois, Robert W Frenck, Wilbur H Chen, Calman A MacLennan, Mark S Riddle, Akamol E Suvarnapunya, Jessica L Brubaker, Karen L Kotloff, Chad K Porter

**Affiliations:** 1 Center for Immunization Research, Department of International Health, Johns Hopkins Bloomberg School of Public Health, Baltimore, Maryland; 2 Enteric Vaccine Initiative, PATH, Washington, District of Columbia; 3 Division of Infectious Diseases, Cincinnati Children's Hospital Medical Center, Cincinnati, Ohio; 4 Center for Vaccine Development, University of Maryland School of Medicine, Baltimore; 5 Bill & Melinda Gates Foundation, London, United Kingdom; 6 F. Edward Hébert School of Medicine, Uniformed Services University, Bethesda; 7 Department of Enteric Infections, Bacterial Diseases Branch, Walter Reed Army Institute of Research, Silver Spring; 8 Global Disease Epidemiology and Control Program, Department of International Health, Johns Hopkins Bloomberg School of Public Health; 9 Division of Infectious Disease and Tropical Pediatrics, Center for Vaccine Development and Global Health, University of Maryland School of Medicine, Baltimore; 10 Enteric Disease Department, Naval Medical Research Center, Silver Spring, Maryland

**Keywords:** *Shigella*, controlled human infection model, human infection studies, challenge studies, methods

## Abstract

*Shigella* causes morbidity and mortality worldwide, primarily affecting young children living in low-resource settings. It is also of great concern due to increasing antibiotic resistance, and is a priority organism for the World Health Organization. A *Shigella* vaccine would decrease the morbidity and mortality associated with shigellosis, improve child health, and decrease the need for antibiotics. Controlled human infection models (CHIMs) are useful tools in vaccine evaluation for early up- or down-selection of vaccine candidates and potentially useful in support of licensure. Over time, the methods employed in these models have become more uniform across sites performing CHIM trials, although some differences in conduct persist. In November 2017, a *Shigella* CHIM workshop was convened in Washington, District of Columbia. Investigators met to discuss multiple aspects of these studies, including study procedures, clinical and immunological endpoints, and shared experiences. This article serves as a uniform procedure by which to conduct *Shigella* CHIM studies.

*Shigella*, a major cause of bacillary dysentery, is an enteric bacterium that can cause inflammatory diarrhea. *Shigella* has long been recognized as a cause of moderate and severe diarrhea and dysentery with a high incidence among children residing in low- and middle-income countries [1, 2]. Recent studies highlight the continued burden of disease and provide data for burden estimates [3–7]. In addition to an estimated 212 438 annual deaths at all age groups [8], children aged <5 years experience nearly 75 million cases of shigellosis each year, leading to stunting and wasting [8–10]. *Shigella* also poses a significant enteric disease threat to deploying military forces and international travelers [11, 12].

The rise of antimicrobial resistance, in addition to the morbidity and mortality caused by *Shigella*, has led to it becoming a priority pathogen for the World Health Organization (WHO) [13]. The WHO's Initiative for Vaccine Research is currently developing preferred product characteristics for *Shigella* vaccines [12], and several candidates are in clinical trials [14]. However, vaccine development to date has been hampered by poor animal models and a lack of immune correlates of protection [15, 16]. The controlled human infection model (CHIM) is a valuable tool that enables early vaccine efficacy evaluation and insights into immunological markers of vaccine-induced immunity [17]. Additionally, CHIMs have been utilized to support vaccine licensure [18, 19], potentially minimizing the time to market, particularly for specific populations such as adult travelers.

The *Shigella* CHIM has been in use to evaluate the efficacy of investigational *Shigella* vaccines since the studies of Shaughnessy and Olsson in 1946 among prison inmates in Joliet, Illinois [20]. Since then, CHIM trials evaluating *Shigella* vaccine candidates have been conducted at several sites in the United States, mostly with *Shigella flexneri* 2a (strain 2457T) [21, 22] and *Shigella sonnei* (strain 53G) [23], but also with wild-type and toxin-minus mutants of *Shigella dysenteriae* type 1 [17, 23–29]. Additionally, trials with 53G have been conducted in Thailand [30, 31]. For CHIM studies evaluating vaccines, there are generally at least 2 phases of the trial: the vaccination phase (often conducted as outpatient) and the challenge phase (conducted as inpatient). Over the decades, there have been refinements in the challenge process; however, procedural and endpoint differences still exist amongst the sites conducting CHIM trials. Recent increasing interest in the *Shigella* CHIM highlights the need for standardization.

A consistent and dependable attack rate in the CHIM is necessary to adequately evaluate vaccines and therapeutics. The attack rate depends on the challenge conditions (fasting, gastric acid neutralization with buffers) [25] and the inoculum dose. It also depends on the primary endpoint definition. The target illness may vary according to whether one is considering prevention of shigellosis and its adverse effects on growth and survival among children living in developing countries, or the prevention of illness experienced by adult travelers where daily function is impacted, is associated with acquisition of multidrug resistance, and may result in persistent gastrointestinal symptoms.

On 27 November 2017, a group of experts was convened by the Bill & Melinda Gates Foundation in Washington, District of Columbia, to discuss standardizing the *Shigella* CHIM. Subsequently, a smaller working group representing the 3 US sites conducting or planning to conduct *Shigella* CHIM (Johns Hopkins University, University of Maryland, and Cincinnati Children's Hospital and Medical Center), as well as experts from other institutions, met in Baltimore, Maryland, on 2 February 2018 to propose a standardized primary endpoint for *Shigella* CHIM (see MacLennan et al in this supplement). To ensure consistency across sites, investigators, and time, here we propose standardized methods for performing *Shigella* CHIMs.

## RECRUITMENT

The recruitment of volunteers for CHIM needs to be done carefully, and concomitant with sufficient education so that potential volunteers understand the rationale behind the study and the risks associated with CHIM. Generally, healthy volunteers are recruited in person; via posted flyers, website postings, radio, television, and newspaper advertisements; or by telephone calls or emails to the clinical trial site. As the challenge phase of these studies is conducted in inpatient facilities, potential volunteers need to have the ability to spend several days away from their normal activities.

### Ethics Approval and Human Subjects Protection

Given the somewhat unusual nature of CHIMs, in that a virulent organism is given to healthy volunteers, a close working relationship with regulatory authorities and ethics oversight committees (institutional review boards [IRBs]) is a must. Controlled human infections have come a long way in terms of the regulatory framework since the first *Shigella* challenges were conducted in the Joliet prison [20]. The ethics of CHIM studies has been discussed and reviewed elsewhere [32–34] and will not be covered here. All CHIM studies today must adhere to good clinical practice (GCP) guidelines, and in accordance with the laws of the country and locality in which they are conducted. The trial must be approved by the appropriate IRB or independent ethics committee (IEC). This includes review of the protocol, informed consent document, other trial material, and any amendments to the protocol. Each IRB/IEC may have specific requirements, which may add variability to the protocol between sites.

## INFORMED CONSENT

Informed consent is an ongoing process that includes the informed consent document but continues throughout the study. The elements of informed consent are defined in the International Council for Harmonisation of Technical Requirements for Pharmaceuticals for Human Use GCP Guidelines. Specific for *Shigella* CHIM studies, there is a need to highlight the requirement for inpatient admission during the time that a volunteer is infectious or ill. The process of the challenge, including the pre- and postfasting, is described. Because primary and secondary endpoints are dependent on stool output, volunteers need to understand that they are expected to collect all stool samples for processing. Additionally, subjects must be told that they may need to self-administer rectal swabs if they are unable to provide a stool sample. The risks of challenge must be clearly delineated and the likelihood of illness explained, including a discussion of the symptoms and treatments such as oral and intravenous (IV) rehydration. The symptoms of shigellosis must be clearly described. This includes the risks associated with acute disease (eg, fever, diarrhea, abdominal pain and cramps, dysentery, dehydration), as well as the risk of chronic sequelae such as irritable bowel syndrome (IBS) and reactive arthritis. Risk-mitigating strategies need to be discussed, including risks from the antibiotics administered. Ciprofloxacin has been the antibiotic of choice for *Shigella* CHIMs. However, significant safety warnings have been raised that need to be shared with volunteers [33–35]. It is worth considering whether an alternative antibiotic can be used that has a better safety profile.

An objective assessment of comprehension is conducted to ensure a basic understanding of the study. One method is to administer a written comprehension assessment. Questions need to include those that focus on the symptoms of shigellosis and postinfection sequelae, the potential need for IV medication or fluid, and the need for admission to an inpatient facility during the challenge phase. Incorrect answers are then discussed with volunteers to reinforce the consent. The necessary percentage correct and the number of attempts permitted should be defined a priori. Our institutions have traditionally set the minimum at 70%–80%. Any volunteer who, in the opinion of the study staff and/or principal investigator, does not understand the study well enough to consider his or her consent truly informed must be excluded.

Compensation for study participation needs to be clearly outlined in the consent form. This compensation should be carefully considered to adhere to an ethical framework without unnecessary inducement [32, 37]. Potential factors to consider include the amount of time a volunteer gives to the study, the risk of illness due to the challenge, and the required confinement. Also, since variability in the duration of the inpatient portion exists depending on center, the time of treatment, and level of shedding of individual volunteers, some adjustments should be considered that do not penalize volunteers for meeting discharge criteria early, but that also fairly compensate those who are asked to spend extra time in the inpatient unit.

## SCREENING OF VOLUNTEERS

Volunteers for challenge studies are generally healthy volunteers, without significant physical or mental health issues. At some sites, those with conditions requiring concomitant medications are excluded. Screening assessments and criteria are geared toward determining general health (Table 1). Given the risks inherent in a CHIM study, and lack of direct benefit to the volunteer, these studies are restricted to adult, nonpregnant volunteers. An upper age limit is generally set to help decrease the risk of adverse events and concomitant illnesses. Some studies limit body mass index (BMI) ranges (as persons with very low or high BMIs have greater risk of other comorbidities). The BMI limits vary by study (often 18–35 kg/m^2^).

**Table 1.** Sample Inclusion/Exclusion Criteria

Inclusion Criteria	
• Healthy, adult, male or female, age (generally 18–50 or 18–45 y)	• Women: Negative pregnancy test with understanding to not become pregnant during the study
• Signed informed consent document.	
• Available for inpatient study and for outpatient follow-up visits	• Completion and review of comprehension test (achieved >70% accuracy)
Exclusion Criteria	
• Clinically significant medical or psychiatric problem by history	• Chronic use of antidiarrheal, anticonstipation, or antacid therapy
• Clinically significant abnormalities on physical examination or screening labs	• History of irritable bowel syndrome or abnormal stool pattern
• Use of steroids or other immunosuppressive and/or immunomodulatory drugs	• Personal or family history of inflammatory arthritis
• Currently pregnant or nursing	• Positive blood test for HLA-B27
• Participation in research involving another investigational product within 30 d before enrollment and during the duration of the study	• History of *Shigella* infection (exclusion duration protocol specific)
• Positive and confirmatory blood test for HBsAg, HCV, HIV-1	• Received previous experimental *Shigella* vaccine or live *Shigella* challenge (exclusion duration protocol specific)
• Clinically significant abnormalities on basic laboratory screening	• Travel to countries with symptoms of travelers' diarrhea where *Shigella* or other enteric infections are endemic within 6 mo prior to enrollment
• Immunosuppressive illness or IgA deficiency (serum IgA <7 mg/dL or limit of detection of assay)	• Occupation involving handling of *Shigella* bacteria
• Current alcohol or drug dependence	• Serum IgG titer ≥2500 to *Shigella* LPS [39, 40]
• Current or prior history of inflammatory bowel disease	• Use of antibiotics within 7 d prior to challenge

Abbreviations: HBsAg, hepatitis B surface antigen; HCV, hepatitis C virus; HIV-1, human immunodeficiency virus type 1; IgA, immunoglobulin A; IgG, immunoglobulin G; LPS, lipopolysaccharide.

Regardless of BMI, volunteers are examined for the ability to obtain venous access if needed. Urine toxicology screening for cocaine, opioids, benzodiazepines, and amphetamines and a history of alcohol and substance use (by volunteer report) may be ascertained to avoid the risk of withdrawal on the unit.

In addition, there are specific screening criteria for *Shigella* CHIM to minimize the potential for long-term sequelae. As undiagnosed IBS may mimic acute diarrheal infections, efforts should be made to exclude volunteers with such endpoint-confounding baseline health conditions. To exclude volunteers with IBS, subjects should have an assessment of stool habit, and a baseline Rome survey, which helps identify functional bowel disorders [41, 42], may be considered. To minimize reactive arthritis risk, volunteers with a history of inflammatory arthritis or who are HLA-B27 positive should be excluded. Some studies prescreen volunteers for preexisting serum antibodies to *Shigella* lipopolysaccharide and exclude those with high titers. A recent study characterizing baseline antibody levels in subjects participating in *Shigella* CHIMs suggests that baseline serum bactericidal titers, opsonophagocytic killing antibody, and IpaB and VirG antibody titers may also contribute to resistance to shigellosis in the challenge model, but this initial observation needs to be confirmed by evaluating more subjects [43].

Ideally, the screening process consists of multiple visits prior to the enrollment of volunteers. This allows study staff to develop a rapport with potential volunteers and allows volunteers to learn about the study and study procedures, and to make truly informed decisions about participating. It also permits the study staff to assess whether a volunteer would be a good candidate for an inpatient setting.

## CHALLENGE INOCULUM PREPARATION FOR *S. FLEXNERI* 2457T AND *S. SONNEI* 53G

Challenge inocula for CHIMs over the last 15 years have been prepared using cell banks produced under GMP (cGMP) from the 2457T strain of *S. flexneri* 2a and the 53G strain of *S. sonnei*. Both strains are phenotypically and genotypically well characterized, including full genomic sequencing [44, 45], and both are pan-antibiotic susceptible. Since both strains are covered under a US Food and Drug Administration drug master file (2457T) or full investigational new drug application (53G), annual stability testing indicates that both have remained stable over time and that they remain susceptible to the antibiotics most commonly used to treat shigellosis: ciprofloxacin and trimethoprim-sulfamethoxazole. Recently, an attempt was made to further standardize the challenge inoculum preparation by using a cGMP lyophilized preparation of the 53G strain (ClinicalTrials.gov identifier NCT028163446), which simplifies the inoculum preparation process, reducing the time required from 3 days to 1 day. The cGMP-produced *Shigella* challenge strains currently available for use in CHIM studies or projected to be available in the near future are outlined in Table 2. A more complete listing of *Shigella* strains used in past CHIMs studies is provided in Porter, et al. [17].

**Table 2.** Available *Shigella* Challenge Strains

*Shigella* Product	Lot No.	Release Date	Volume (mL) per Tube and CFU/mL	Strain Source		
				WRAIR	PATH	CVD
*S. flexneri* 2a strain 2457T (PCB) [21]	1617	25 Jan 2011	1.0 1.8 × 10^8^	Yes	Yes	Yes
*S. flexneri* 3a strain J17B (PCB)	1654	7 Apr 2011	1.0 1.5 × 10^8^	Yes	Yes	No
*S. sonnei* strain 53G [46] lyophilized	1794	14 Mar 2014	2.0 2.0 × 10^9^	Yes	No	No

It is anticipated that good manufacturing practice master cell bank and PCB for *S. flexneri* serotypes 6 (strain CCH060) and 1a (strain to be determined) will be produced by WRAIR in 2019–2020. Lyophilized preparations of *S. flexneri* serotypes 2a (2457T) and 3a (J17B) are projected for 2019 at WRAIR. Strain J17B has yet to be evaluated in a controlled human infection model. With eventual production of the *S. flexneri* 6 and 1a cell banks, challenge strains would be available for the *Shigella* serotypes most commonly associated with moderate to severe diarrhea in the recent Global Enteric Multicenter Study [47].

Abbreviations: CFU, colony-forming units; CVD, Center for Vaccine Development, University of Maryland School of Medicine; PCB, production cell bank; WRAIR, Walter Reed Army Institute of Research.

**Figure 1.**
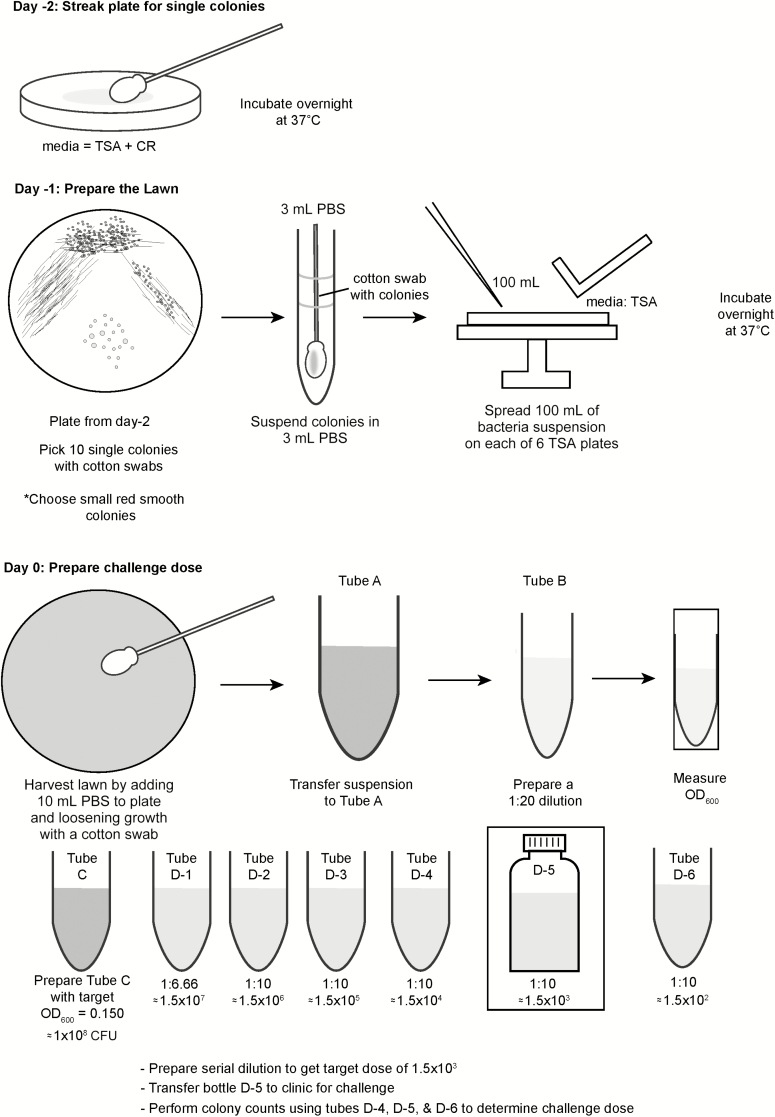
Inoculum preparation procedure. Abbreviations: CFU, colony-forming units; CR, Congo Red Dye; OD_600_, optical density 600 nm; PBS, phosphate-buffered saline; TSA, trypticase soy agar.

For freshly harvested cells (Figure 1), approximately 48 hours prior to volunteer dosing, the cGMP is plated onto 5 plates containing trypticase soy agar (TSA), to which Congo red dye (0.01%) has been added. The plates are streaked for isolation to yield well-separated colonies and then incubated at 37°C ± 1°C for 22 ± 2 hours. Congo red–positive colonies are tested for agglutination with commercial *Shigella* serotype-specific antiserum and then 10 well-isolated Congo red–positive colonies are picked and used to prepare a bacterial suspension in 3 mL of phosphate-buffered saline (PBS). This suspension is used to inoculate multiple TSA plates to yield confluent growth (~6 plates for 30 volunteers, plus 2–3 extra plates as backup in case of individual plate contamination). After growth for 22 hours at 37°C ± 1°C, the TSA plates are visually screened for purity, growth is rechecked for agglutination in antiserum, and cells are harvested in sterile PBS (pH 7.4). For this step, 10 mL of PBS is added to each TSA plate and a sterile cotton swab is used to gently loosen the bacterial growth from the surface of the TSA plate. The bacterial suspension from each plate is collected with a sterile pipette, pooled, and diluted in PBS to the target dose (colony-forming units [CFU]/mL) based on target optical density 600 nm (OD_600_) values. The diluted preparation is administered to challenge study volunteers within 4 hours of preparation.

For CHIM using lyophilized bacteria, the challenge inoculum is prepared on the day of volunteer dosing. One multidose vial is removed from −80°C storage and thawed on ice for 30 minutes. To reconstitute the bacteria, sterile water for injection is aseptically added to the vial and the vial then incubated on ice for another 15 minutes with intermittent swirling to mix the contents. Once fully reconstituted, the volunteers are dosed within 4 hours. To prepare the challenge doses, the reconstituted bacterial suspension is diluted in normal saline, based on OD_600_ target values, to the desired dose (CFU/mL).

For dosing with either the lyophilized or frozen inoculum, 1 mL of the adjusted suspension is added to 30 mL of saline or bicarbonate buffer (13.35 g of sodium bicarbonate in 1000 mL of sterile water) for each subject for oral administration. The bacterial suspension is plated on TSA before and after challenge to estimate the administered inoculum dose. The inoculum may also be plated on Congo red agar to ensure the majority of colonies have retained the invasion plasmid.

Key elements in assuring the reproducibility of the inoculum preparation process are training of laboratory staff in all study processes and procedures; quality control on all media, reagents, and equipment; and conducting a sufficient number of practice runs to document that the inoculum target dose can be met. It is also important to have all practice and actual inoculum preparation, including colony counts and challenge dose calculations, monitored by a representative of the research pharmacy of the institution performing the CHIM or a similar independent group.

An important consideration for future CHIMs studies might be to consider genomics and transcriptomic analysis of the challenge strain before dosing and after recovery from subjects to determine the extent to which passage through the host and interaction with the gut microbiome may serve to alter the strain or lead to the expression of antigens that might have vaccine potential. Theses studies have occasionally been done, but the field might benefit from a more systematic approach on this, taking advantage of the more controlled nature of the CHIM design.

## ADMISSION TO INPATIENT FACILITY

CHIM studies can be conducted in a variety of inpatient settings ranging from hospital beds on inpatient general medicine wards, to specialized clinical research units within hospitals, to stand-alone inpatient units. Volunteers may be housed in individual rooms, dorm-like settings where several are in a room, or in a large open unit. Critically important, the facility in which the volunteers are housed needs to be equipped to prepare or administer the challenge inoculum, manage volunteers sick with shigellosis, and to collect and properly process stool and immunologic samples.

In addition to staff experienced in handling samples, appropriate medical and nursing care needs to be available. Nursing care should be present 24 hours a day, with sufficient staff available to handle multiple acutely ill volunteers simultaneously. For example, at 1 center, during times of peak illness, a minimum of 1 staff person is scheduled for every 5–6 volunteers during the evenings and night. During the day, when more examinations are performed and samples are processed and shipped, more staff members are present (including clinicians). When volunteers are not sick, and fewer samples need processing, the staffing level can be reduced (keeping a minimum of 2 staff on at all times).

Staff should be trained on the study protocol and procedures prior to admission. Staff should be able to comfortably place IV catheters and administer IV fluids. Temporary staff, if utilized, should complete human subjects protection training prior to working on a clinical study. Necessary medication should be immediately available: if there is no 24-hour pharmacy at the facility, the facility should be appropriately stocked with antibiotics, antiemetic medication (including IV antibiotics and antiemetics), antipyretics, and allergy medication, as well as other medication that may be necessary during a period of confinement (eg, nicotine patches). Monitoring equipment and the ability to administer IV fluids needs to be present, together with a laboratory facility to process blood and stool samples, and the ability to safely dispose of infectious biological samples.

Before admission, volunteers should be informed of the rules of the inpatient facility and given a list of impermissible items including weapons, cigarettes (and e-cigarettes), tobacco, alcohol, scissors/knives, matches, lighters, outside food or drink, and large amounts of money. All items brought to the unit (including clothing) should be examined carefully to exclude prohibited items. At some sites, volunteers are given medical scrubs to wear (cloth or disposable paper scrubs) while on the unit, so there is minimal need for personal clothing. At other sites, volunteers are given a choice of personal clothing or scrubs. Unnecessary items should be secured in a limited-access location. In addition, volunteers are allowed to bring toiletries, electronic devices, and entertainment materials (games, books, etc). Prescription medication (eg, oral contraceptive pills or antihypertensives) is stored with the other medications for the study and administered to the volunteer at the appropriate time.

Generally, volunteers are admitted at least 1 day prior to receipt of the *Shigella* inoculum to ensure all prechallenge procedures, including laboratory testing, are completed prior to challenge. This day also allows for acclimation of the volunteers to the unit and each other and a chance for the staff to observe volunteers for potential behavioral problems. At admission, volunteers are evaluated (vital signs, medical history, physical examination, serum or urine pregnancy testing) to ensure no exclusionary conditions have arisen. Laboratory (clinical and research) assays are collected to ensure available baseline parameters. Stool samples, if produced prechallenge, are collected for baseline microbiological and immunological assessment. The growth of enteric coliforms prior to challenge is a reassuring sign of normal bowel health.

## COLLECTION, EVALUATION, AND PROCESSING OF STOOLS

**Figure 2.**
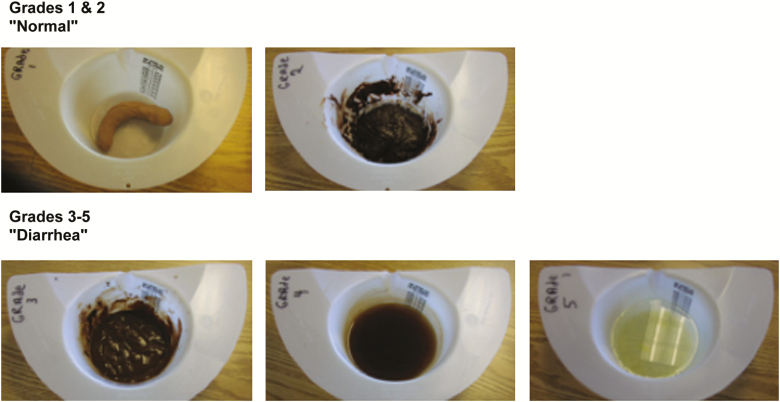
Stool grading.

After admission, subjects are required to collect all stools passed using stool collection kits that fit over toilets (eg, Commode specimen collection system, Fisher Scientific). The kit comes with a lid that can be placed on the sample for transport to the laboratory. Volunteers are encouraged to write their study numbers, initials, and time collected on the kit. Stool samples are brought to the nurses' station or laboratory where study staff inspect the contents of the stool container and grade the stool consistency according to Figure 2. Staff weigh all stools and record the stool grade, weight (as a proxy for volume), and the presence of gross blood or mucus on a stool record sheet. When gross blood is visualized, its presence is confirmed using a test for occult blood. Staff then place a sample of the stool into sterile containers for bacteriologic culture and other assays as required by the clinical protocol and the immunology and microbiological testing plans. In some cases, prepared media (eg, RNA-Later for transcriptomic studies or protease inhibitors for assessment of fecal antibody responses and intestinal markers of inflammation postchallenge) in which to place a sample of the stool for future assays may be used. The remaining stool is treated with bleach either before or after disposal in a commode or hopper.

## STOOL CULTURE FOR CHALLENGE ORGANISM

Postchallenge stools should be cultured daily until the challenge organism has been cleared. Both qualitative and quantitative cultures can be done. Stool stored in sterile containers are kept at 4°C until cultured. For qualitative cultures, stool is streaked onto a MacConkey agar plate, and a *Salmonella/Shigella* agar plate with sterile loops. Hektoen enteric agar may also be used. After an incubation of 14–24 hours at 37°C, 5 *Shigella*-appearing colonies are picked (preferentially from the MacConkey agar) and individually tested against *Shigella* antisera for agglutination. For quantitative cultures, measured amounts of stool are serially diluted in sterile saline and plated on MacConkey agar and a selective media to determine the CFUs of the challenge strain per gram of stool. Identification of the challenge strain on these plates is based on agglutination of up to 10 colonies in *Shigella* antisera or immunoblotting of full-plate colony lifts. Quantitative polymerase chain reaction (PCR) may be an alternative to culture-based methods to monitor fecal shedding of the challenge strain. Recent field and CHIM data [4, 48] indicate that the level of *Shigella* being shed is associated with the development of clinical illness, so the determination of shedding levels following challenge may give an additional secondary endpoint for assessing the impact of preventive interventions, such as vaccines.

### Collection and Processing of Blood Samples

Each protocol is designed to answer specific questions about the immune response in vaccinated and unvaccinated volunteers before and after challenge. The type of assays and their frequency will depend on the nature of the specific hypotheses to be tested. These samples can be serum, whole blood, or peripheral blood mononuclear cells.

### Prechallenge Verification of Eligibility

Eligibility is reassessed the morning of challenge using a focused medical history to identify the onset of symptoms that could indicate a new infectious illness (eg, anorexia, malaise, cramps, headache, and vomiting). A focused physical examination is performed at the discretion of the physician according to the nature of a volunteer's solicited or unsolicited complaint. The physician reviews each volunteer's standardized source documents on which all stools and vital signs are documented to assess for new occurrence of loose stools or fever and ensures that all women have had a negative pregnancy test during the preceding 24 hours. The physician then provides written verification that all eligibility criteria have been met.

## CHALLENGE PROCESS AND INOCULUM ADMINISTRATION

On the day of challenge, volunteers fast for 90 minutes to facilitate consistency of attack rate. Approximately 1 minute prior to challenge, the volunteers drink 100–120 mL of bicarbonate buffer. For the challenge, volunteers drink a 30-mL solution of sterile water or bicarbonate buffer containing the *Shigella* challenge strain (~1500 CFU, with an acceptable range of 1500–2000 CFU). Volunteers are observed closely during ingestion of the buffer and inoculum to ensure that both are fully consumed. Subjects are directly observed for 30 minutes for any immediate adverse reactions. After a 90-minute postchallenge fast, volunteers are again permitted to eat and drink. Some institutions limit certain food and beverages after challenge, such as dairy products and caffeine, while others do not.

### Clinical Evaluation on the Inpatient Unit

Volunteers are monitored carefully throughout their inpatient stay. Vital signs and oral temperature are measured regularly (at least 3 times daily) by clinical staff who remain on the unit 24 hours a day. A physician is available onsite or by telephone or pager at all times. Volunteers are interviewed daily by a physician to solicit specific symptoms (anorexia, malaise, abdominal pain or cramps, headache, fever, tenesmus, and vomiting), which are recorded on a standardized form and graded in severity. As symptoms and severity of symptoms are important in the outcome definitions, training and standardization of symptom collection and characterization are important. The clinician needs to verify the specificity of symptoms and be sure to annotate the severity of individual symptoms based both on observation and patient reports. Under and overendorsement needs to be addressed to assure accurate and representative recording of symptoms. Clinic operating manuals and training are important.

### Clinical Management of Volunteers Who Develop Loose Stools

Volunteers who develop loose stools (grades 3–5) are encouraged to maintain good oral fluid intake. Oral intake and stool output are carefully monitored. Procedures for the use of IV fluids (lactated Ringer solution or similar fluid) are described in site procedures. Generally, IV fluid is administered when a volunteer is unable to keep up with the fluid lost due to diarrhea via oral intake, has a high fever, or shows signs or symptoms of dehydration (which can occasionally occur without frank diarrhea).

### Antibiotic and Antipyretic Therapy Following Challenge

All challenge strains are documented to be susceptible to the antibiotics used to treat volunteers. If volunteers do not meet early treatment criteria, 5 days (~120 hours postinoculation) after challenge all volunteers receive a 3- to 5-day antibiotic course. Generally, ciprofloxacin 500 mg by mouth twice a day is started. Trimethoprim-sulfamethoxazole (160 mg/800 mg) every 12 hours or ampicillin 500 mg by mouth every 6 hours for 5 days can be substituted in the event of an allergy or adverse reaction to ciprofloxacin. Given increasing concerns about the safety of fluoroquinolones [35, 36], investigators may wish to consider an alternative first-line antibiotic. Also, as loperamide has been shown to decrease time to diarrhea resolution, consideration should be given as to whether concomitant treatment together with antibiotic should be routinely used [49, 50].

Early antibiotic therapy is initiated if the volunteer meets the primary endpoint of shigellosis and continues to feel ill or has a fever ≥39.0°C, or for other indications as deemed appropriate by the investigator. In theory, infection could relapse after antibiotic treatment is complete, as has occurred with *Campylobacter jejuni*. However, this has never been observed with either wild-type *Shigella* or with live attenuated *Shigella* vaccines treated with ciprofloxacin. If retreatment with antibiotics is needed because the first course did not eradicate the vaccine strain, susceptibility testing should be performed, and a second course of antibiotics administered.

### Criteria for Discharge

As a precaution against spread of *Shigella* from volunteers on the inpatient unit to the community, investigators require that all volunteers eradicate *Shigella* prior to discharge from the inpatient unit. Volunteers are treated regardless of whether they are found to be excreting *Shigella* after challenge. A volunteer is eligible for discharge from the inpatient facility once he or she has ingested at least 2 doses of antibiotics and has had at least 2 stools with consecutive negative cultures for the challenge organism with clinical symptoms resolved or resolving. Generally, volunteers are eligible for discharge 7–8 days after challenge (2–3 days after commencement of antibiotics). Volunteers who meet early treatment criteria can be discharged early.

### Management of Early Termination From the Challenge Study

During the screening process, volunteers are repeatedly educated about the importance of completing all study procedures. Nonetheless, from time to time a volunteer decides to leave the study or the investigator decides to discharge volunteers from the unit before the criteria for discharge have been fulfilled. When this occurs, the volunteer is counseled about the risk of transmission of the strain to close contacts and asked to read and sign a form explaining the risks and strategies to mitigate these risks. A 1-g oral dose of ciprofloxacin is given on the unit, as this has been shown to be 100% effective in eliminating infection and symptoms of shigellosis not caused by *S. dysenteriae* type 1 [51–53]. The volunteer is sent home with additional tablets of ciprofloxacin to complete the course of therapy at home and is asked to make the protocol-specific outpatient follow-up visits. At a minimum, efforts should be made to follow a subject for safety for at least 28 days following the challenge inoculation.

### Follow-up Visits

After discharge from the inpatient unit, volunteers are usually required to make follow-up visits to the clinical site over a 28- to 42-day period to provide specimens of blood to measure immune responses and stool for culture, immunology, or microbial testing. They are given sterile screw-top containers, vials containing transport media, and cooler bags with a cold pack for collection of the required outpatient stool specimens. Volunteers swab their stool sample and place the swab into the vial containing transport media. The whole stool and stool vial are brought to the laboratory within a certain number of hours in the cooler bag. At each follow-up visit, clinical histories are taken to record recurrent shigellosis, signs or symptoms of postinfectious reactive arthritis, hospitalizations, physician visits, or other serious medical concerns. The incidence of serious adverse events and adverse events of special interest should be assessed for at least 6 months after challenge. Volunteers who develop adverse events of special interest should be followed for resolution or stabilization of the symptoms. If necessary, they should be provided with referrals and access to appropriate specialists. In addition, the development of postinfectious irritable bowel syndrome can be assessed by repeating the Rome III survey. This can be conducted by a phone interview.

## CLINICAL ENDPOINTS

In addition to the primary clinical endpoint (see MacLennan et al in this supplement), there are numerous secondary endpoints that are important in differentiating study groups (eg, vaccine effect) (Table 3). These are described in detail below and include endpoints associated with the frequency and/or volume of loose stool output, presence and severity of nondiarrheal signs and symptoms, and a composite severity score that encompasses both stool output and clinical signs and symptoms. Comparison of continuous and/or ordinal data are made using parametric (eg, *t* test or analysis of variance) or nonparametric (eg, Kruskal-Wallis or Mann-Whitney *U* test) methods as appropriate. For nominal data, proportions are compared using pairwise Pearson χ ^2^ or Fisher exact test. The time to event data are compared using the product-limit method. If >2 groups are being compared, appropriate post hoc pairwise (as applicable) comparisons will be made with appropriate α adjustments if the omnibus null hypothesis is rejected. All point estimates for percentages should be accompanied by 95% confidence intervals (either exact or asymptotic estimates based on sample size).

**Table 3.** Secondary Clinical Endpoints

Secondary Clinical Endpoints	
• Percentage of volunteers with diarrhea	• Maximum 24-h stool output
• Mean/median time to onset of diarrhea	• Total loose stool output
• Mean/median duration of diarrhea	• Percentage of volunteers with nausea, vomiting, anorexia, gas, or abdominal pain/cramps rated as moderate to severe
• *Shigella* disease severity score postchallenge	• Percentage of subjects with dysentery
• Percentage of subjects with fever	• Percentage of subjects requiring intravenous fluids
• Percentage of subjects requiring early antibiotic therapy	

### Maximum 24-Hour Stool Output

The maximum number of loose stools in a 24-hour period is calculated by counting the frequency of loose stools in each 24-hour period. The 24-hour period is rolling and starts with the passing of each loose stool. Similarly, the maximum volume of output in a 24-hour period is estimated based on stool weight (assuming 1 mL = 1 g) and is similarly calculated with a rolling 24-hour interval. By-group estimation of the mean (standard deviation) and/or median (interquartile range) is calculated.

### Percentage of Volunteers With Diarrhea (All Severities)

The percentage of volunteers with diarrhea (any severity and for each level of severity separately) is determined based on the maximum 24-hour stool output as follows: mild diarrhea, 2–3 loose stools or <400 g/grade 3–5 loose stools per 24 hours; moderate diarrhea, 4–5 loose stools or 400–800 g loose stools per 24 hours; severe diarrhea, ≥6 loose stools or >800 g loose stools per 24 hours. Initial estimates are calculated (and comparisons made) with either frequency or volume. Subsequent analyses may stratify volunteers meeting the definition by volume or by frequency. This will also be done for moderate to severe diarrhea endpoints separately.

### Total Loose Stool Output

The total frequency and weight (assuming 1 mL = 1 g) of loose stools is assessed by counting the number of loose stools and by summing the weight of each loose stool by volunteer following inoculation. This can include 3 separate estimates as follows: (1) all loose stool samples postinoculation; (2) only loose stool samples postinoculation prior to receipt of antibiotic treatment; (3) all loose stool samples postinoculation that the principal investigator felt were associated with the inoculation.

### Percentage of Volunteers With Nausea, Vomiting, Anorexia, Gas, or Abdominal Pain/Cramps Rated as Moderate to Severe

The number and percentage of volunteers with any symptoms related to the *Shigella* challenge will be calculated from the listing of symptoms that occurred during the inpatient phase of the study.

### Mean/Median Time to Onset of Diarrhea

Time to onset of diarrhea (in hours) is calculated by determining the number of hours it takes for a volunteer to produce the first grade 3–5 stool that contributes to meeting the diarrhea definition after administration of the challenge strain. Volunteers whose first loose stool occurs after the receipt of the antibiotic will be evaluated by an independent board to determine if their loose stools are related to the challenge strain.

### Mean/Median Duration of Diarrhea

The duration of diarrhea (in hours) will be assessed based on the number of hours from the first and last loose stool that are part of a diarrheal episode. The duration of diarrhea for volunteers with no diarrhea will be handled in 2 separate analyses as follows: (1) included in the overall analysis with a value of “0” for the duration of the diarrheal episode; or (2) excluded from the analysis.

### Shigella Disease Severity Score Postchallenge

Porter et al [54] recently published a *Shigella* disease severity index. A *Shigella* disease severity score will be calculated for each volunteer and compared across groups.

## CONCLUSIONS

*Shigella* CHIMs are useful for evaluating potential therapeutics and vaccines. As this organism is increasingly recognized as a cause of morbidity and mortality, a vaccine is now a global health priority. The *Shigella* CHIM allows for rapid evaluation of vaccine efficacy for up- or down-selection of vaccine candidates. Opportunities have arisen to validate the model as a predictor for vaccine efficacy in the field. For example, the streptomycin-dependent *S. flexneri* 2a vaccines conferred significant protective efficacy in both the CHIM [22] and in field trials [55]. The protective efficacy of *S. sonnei* against homologous rechallenge (78%) [56] was comparable to efficacy provided by natural infection in the field [57].

To compare potential vaccine candidates using the CHIM, CHIM standardization across institutions and over time is necessary. With time, further refinement of the CHIM may occur, for example, different fasting durations may impact attack rates. In addition, alternative antibiotics, the use of loperamide as an adjunct to antibiotics, and the use of different discharge criteria (potentially with the addition of quantitative PCR) may be evaluated.

Depending on the vaccine candidates tested, and vaccine efficacy observed, multiple cohorts, potentially across multiple institutions may be needed to ensure sufficient power. In addition, given the constraints of time, volunteer availability, and cost, it may not be possible to adequately power studies to reach an unambiguous determination. In this situation, an indeterminate result might be sufficient to allow a vaccine to advance to further efficacy evaluation and to support licensure. This result might be supported by other evidence of positive vaccine impact, such as a favorable reduction in disease severity score.

## Notes

***Disclaimer.*** The views expressed in this article do not necessarily reflect the official policy or position of the Uniformed Services University, Department of the Navy, Department of the Army, Department of Defense, nor the US government. This is a partial US government work. There are no restrictions on its use. Three of the authors (M. S. R., A. E. S., and C. K. P.) are employees of the US government. This work was prepared as part of official duties. Title 17 U.S.C. §105 provides that “[c]opyright protection under this title is not available for any work of the United States government.” Title 17 U.S.C. §101 defines a US government work as a work prepared by a military service member or employee of the US government as part of that person's official duties.

***Supplement sponsorship***. This supplement is sponsored by the Bill & Melinda Gates Foundation.

***Potential conflicts of interest.*** C. K. P. has received collaborative research and development funding from LimmaTech Biologics outside this work. A. E. S. has received grants and nonfinancial support from PATH outside this work. K. R. T. has received grants from LimmaTech Biologics outside this work. W. H. C. has received grants from the Bill & Melinda Gates Foundation and from Institut Pasteur outside this work. All other authors report no potential conflicts of interest. All authors have submitted the ICMJE Form for Disclosure of Potential Conflicts of Interest. Conflicts that the editors consider relevant to the content of the manuscript have been disclosed.
